# Gradient-echo magnetic resonance imaging study of pancreatic iron overload in young Egyptian beta-thalassemia major patients and effect of splenectomy

**DOI:** 10.1186/1758-5996-2-23

**Published:** 2010-04-15

**Authors:** Randa M Matter, Khalid E Allam, Amany M Sadony

**Affiliations:** 1 Department of Pediatrics, Faculty of Medicine, Ain Shams University, Cairo 11566, Egypt; 2 Department of Diagnostic Radiology, Faculty of Medicine, Ain Shams University, Cairo 11566, Egypt

## Abstract

**Background:**

Thalassemic patients suffer from diabetes mellitus secondary to hemosiderosis.

**Aims:**

The study aimed to evaluate pancreatic iron overload by T2*-weighted Gradient-echo magnetic resonance imaging (MRI) in young beta-thalassemia major patients and to correlate it with glucose disturbances, hepatic hemosiderosis, serum ferritin and splenectomy.

**Methods:**

Forty thalassemic patients (20 non diabetic, 10 diabetic, and 10 with impaired glucose tolerance) were recruited from Pediatric Hematology Clinic, in addition to 20 healthy controls. All patients underwent clinical assessment and laboratory investigations included complete blood count, liver function tests, serum ferritin and oral glucose tolerance test (OGTT). A T2*-weighted gradient-echo sequence MRI was performed with 1.5 T scanner and signal intensity ratio (SIR) of the liver and the pancreas to noise were calculated.

**Results:**

Significant reduction in signal intensity ratio (SIR) of the liver and the pancreas was shown in thalassemic patients compared to controls (P < 0.0001), Thalassemic patients with abnormal glucose tolerance; including diabetics and thalassemics with impaired glucose tolerance; displayed a higher degree of pancreatic and hepatic siderosis compared to thalassemics with normal glucose tolerance or controls (P < 0.001, P < 0.0001). Splenectomized thalassemic patients had significantly lower SIR of pancreas compared to non splenectomized patients (P < 0.05). A strong correlation was present between hepatic and pancreatic siderosis in studied patients (P < 0.001).

**Conclusions:**

pancreatic siderosis can be detected by T2* gradient-echo MRI since childhood in thalassemic patients, and is more evident in patients with abnormal glucose tolerance. After splenectomy, iron deposition may be accelerated in the pancreas. Follow up of thalassemic patients using pancreatic MRI together with intensive chelation therapy may help to prevent the development of overt diabetes.

## Introduction

Thalassemic patients affected by systemic iron overload, often die owing to iron induced failure of vital organs such as heart and liver. Impairment of the endocrine and exocrine function of the pancreas is a common complication in patients with beta-thalassemia major [1,2]. The incidence of impaired glucose tolerance and diabetes in thalassemia major patients varied from 9% to 15% depending on the age of assessment, the intensity of chelation, transfusion and related patient compliance [3,4]. The etiology of diabetes in β-thalassemia is increased peripheral resistance to insulin and direct toxic effect of excess iron in the acinar and beta cells of pancreas resulting in insulin deficiency [5]. Structural abnormalities of the pancreas have been described on sonography [1,6].

Magnetic resonance imaging has emerged for the non-invasive quantification of hepatic iron [7,8]; it represents a reliable method for assessing iron overload in various tissues, not easily accessible with other techniques [9,10]. Despite extensive research on MRI of hepatic and cardiac iron overload, there have been limited data on the MRI of pancreas in patients with β-thalassemia major [11-13]. Moreover, the relation between pancreatic and hepatic siderosis and, between MRI of the pancreas and diabetes has not been documented in medical literature [13]. Very few studies have evaluated the effect of age on pancreatic siderosis and compared MRI data from younger and older patients with thalassemia [12].

The objective of this study was to evaluate pancreatic iron overload by the non-invasive T2*-weighted gradient-echo MRI (T2*GRE) in children and adolescents with beta thalassemia major and to correlate it with glucose disturbances, splenectomy, serum ferritin, and hepatic siderosis as expressed by signal intensity ratio.

## Subjects and Methods

This cross-sectional study comprised 40 Egyptian transfusion dependant β-thalassemia major patients (24 males and 16 females) recruited from the Hematology Clinic, Children's Hospital, Ain Shams University, Cairo, Egypt. The study was approved by the Local Ethical Committee. All patients were diagnosed as β-thalassemia major based on clinical and hematological evaluation, age ranged between 8-18 years (13.58 ± 3.57). Patients were clinically evaluated. All patients were on regular blood transfusion (15 ml packed RBCs/Kg body weight) at 2-4 weeks interval to keep their hemoglobin at a level of 6-8 g/dl before each transfusion. Patients were receiving chelation therapy with subcutaneous deferoxamine mesylate (Desferal, Ciba Geigy, Basel, Switzerland) (30 to 40 mg/kg per day) or oral Deferiprone (50-75 mg/kg/day). Compliance to chelation therapy varied among patients from two to five days per week. Those having acute systemic infection were temporarily excluded from the study to omit the influence of infection on ferritin. Splenectomy has been performed in 32/40 patients (80%). The studied patients were categorized according to the results of OGTT into 20 patients with thalassemia major with normal glucose tolerance (NGT) and 20 patients with abnormal glucose tolerance. Those with abnormal glucose tolerance were further subdivided into 10 patients with impaired glucose tolerance (IGT) and 10 patients with overt diabetes (8 were known diabetics on insulin therapy and 2 were diagnosed during the study by OGTT). Their data are shown in (Table 1). Twenty age and sex matched healthy children and adolescents, 12 males and 8 females; aged 13.6 ± 2.39 years with normal hemogram, liver function tests, serum ferritin and OGTT served as control group. Informed consent was obtained before the study from all subjects or their legal guardians.

**Table 1:** Clinical and laboratory data of thalassemic patients with normal glucose tolerance, impaired glucose tolerance and diabetes.

Parameters	Normal Glucose Tolerance(20)	Impaired Glucose Tolerance (10)	Diabetics (10)
Male/Female	14/6	5/5	6/4

	**Mean ± SD**	**Mean ± SD**	**Mean ± SD**

Age (yrs)	13.15 ± 3.56	14.80 ± 3.12	14.90 ± 2.69

Age of diagnosis (ms)	8.5 ± 6.53	7.80 ± 3.22	6.70 ± 2.50

Duration of disease(yrs)	12.42 ± 3.52	13.21 ± 3.3	14.34 ± 2.66

Duration of chelation (yrs)	5.80 ± 3.07	5.55 ± 4.31	5.90 ± 2.96

Hb (g/dL)	7.46 ± 1.24	7.53 ± 1.23	7.39 ± 0.86

AST (IU/L)	64.80 ± 44.06	86.89 ± 40.07	74.80 ± 41.52

ALT (IU/L)	66.40 ± 53.74	107.50 ± 84.53	67.70 ± 27.77

Serum ferritin (ng/ml)	1999.90 ± 932.95	3223.60 ± 2188.23	3438 ± 1327.60

FBG (mg/dL)	82.40 ± 7.16	107.8 ± 16.4	145.30 ± 29.50

PP (mg/dL)	120.80 ± 9.55	175.2 ± 17.9	236.80 ± 29.81

Frequency of blood transfusion (wk interval)	3.05 ± 0.6	3.2 ± 0.63	3.4 ± 0.84

SD, standard deviation; Hb, Hemoglobin; AST, aspartate aminotransferase; ALT, alanine aminotransferase; FBG, fasting blood glucose; PP, postprandial blood glucose

Laboratory investigations: Complete blood count was performed using coulter B66, Miami, Florida, USA, serum ferritin on immulite instrument (Diagnostic products corporation 5700 West 96 St. Los Angeles, USA), fasting blood glucose(FBG), postprandial blood glucose (PP), liver function tests including aspartate aminotransferase (AST), alanine aminotransferase (ALT) using Synchron CX9 autoanalyzer (Brea, California, USA). Oral glucose tolerance test (OGTT) was performed as described by WHO [14], using a glucose load containing the equivalent of 1.75 g/kg body weight to a maximum of 75 g anhydrous glucose dissolved in water. For evaluation of OGTT test, WHO criteria were used: (normal glucose tolerance: venous plasma glucose 2 hours after glucose load <140 mg/dl; impaired glucose tolerance: 140-199 mg/dl; diabetes mellitus: ≥200 mg/dl). Diagnosis of diabetes mellitus was also considered when fasting plasma glucose was ≥126 mg/dl on at least two occasions [14].

### T2*MRI imaging

#### Technique

All MRI examinations were performed with a 1.5 T scanner (Gyroscan ACS NT; Philips Medical Systems, Best, the Netherlands). Breath-hold gradient-echo sequence was performed using a body coil to avoid signal drop-off [15] and to ensure the highest uniformity in the signal-to-noise ratio throughout the scanned volume. The pulse sequence applied was T2-weighted gradient-echo sequence TR/TE = 120/15 msec with 25 slices to cover the whole abdomen, including the liver and pancreas in the scanning field. The patients were laid supine on the scanner table. It was obtained in the axial plane, flip angle 20°; with slice thickness 7 mm with an interslice gap of 1 mm, image matrix 256 × 256 pixels and a field of view of 370 (350-400 mm). These MRI protocol was based on literature data[15,16].

#### Image interpretation

The signals intensity (SI) of the hepatic parenchyma was considered as the average of three signals intensity measurements obtained at circular regions of interest (ROIs) that measured 1-2 cm^2^, always greater than 35 pixels and were located in the right lobe of liver, away from vascular structures or breathing artifacts and far from organ boundaries. We measured the signal intensity of the pancreatic parenchyma in two ROIs measuring 1-2 cm^2 ^far from the boundaries of the organ to avoid apparent blood vessels and bile ducts and estimated the average. Signal to noise ratio was calculated.

### Statistical Analysis

Analysis of data was performed using SPSS (version 15). Comparison between 2 groups of patients was made using Student's t-test for parametric measures and Mann-Whitney U test for non parametric measures. Comparison between multiple groups of patients was made using ANOVA test and Kruskal-Wallis test. Differences in proportions between 2 groups were evaluated using Chi-Square test and Fisher's exact test. Spearman's rank correlation coefficient was used to measure how SIR and other parameters were correlated. P value < 0.05 was considered the cut-off value for significance.

## Results

Thalassemic patients with NGT and abnormal glucose tolerance were comparable as regards mean age, age at diagnosis, duration of disease, Hb% and liver enzymes (P > 0.05). There were significantly higher mean fasting blood glucose (FBG), postprandial blood glucose (PP) and mean serum ferritin levels in patients with abnormal glucose tolerance compared to non diabetic patients (P < 0.05) (Table 2). The two patient groups were also comparable as regards the distribution of sex and splenectomy using Fisher's exact test (P > 0.05).

**Table 2:** Comparison between thalassemic patients with normal and abnormal glucose tolerance as regards clinical and laboratory data

Parameters	Normal glucose tolerance(20)		Abnormal glucose tolerance (20)		t *	P	Sig.
				
	Mean ± SD		Mean ± SD				
Age (yrs)	13.15 ± 3.56		14.85 ± 2.83		1.67	0.10	NS

Age at diagnosis (ms)	12.30 ± 18.63		7.20 ± 2.78		1.21	0.23	NS

Duration of chelation (yrs)	5.80 ± 3.07		5.73 ± 3.60		0.07	0.94	NS

Hb (gm/dl)	7.46 ± 1.24		7.61 ± 1.4		0.4	0.33	NS

FBG (mg/dl)	82.40 ± 7.16		138.75 ± 30.39		8.07	<0.0001	**HS**

PP (mg/dl)	120.80 ± 9.55		212.50 ± 47.58		8.45	<0.0001	**HS**

	**Median**	**IQR**	**Median**	**IQR**	Z**	**P**	

Serum ferritin (ng/ml)	1704.5	1330-2000	3251	3050-4300	-3.03	0.002	**HS**

Abnormal glucose tolerance includes impaired glucose tolerance and diabetes;*Student t test **Mann-Whitney test; SD, standard deviation; IQR, interquartile range (25-75th percentile range); Hb, Hemoglobin; FBG, fasting blood glucose; PP, postprandial blood glucose

**Figure 1:**
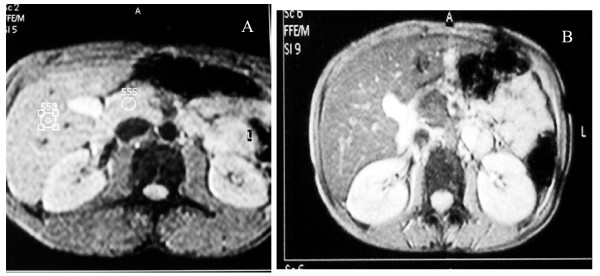
T2*-weighted GRE image of (A) normal subject and (B) β-thalassemia major patient with adequate chelation: (A) T2*-weighted GRE image (TR/TE/FA 120/15/20) of 18 yr-old normal male subject with normal signal intensity in the liver and the pancreas. (B) T2*-weighted GRE image (TR/TE/FA 120/15/20) of 13 yr-old male with β-thalassemia major with adequate chelation showing normal signal intensity in the liver and the pancreas (TR, repetition time; TE, echo time; FA, flip angle).

**Figure 2:**
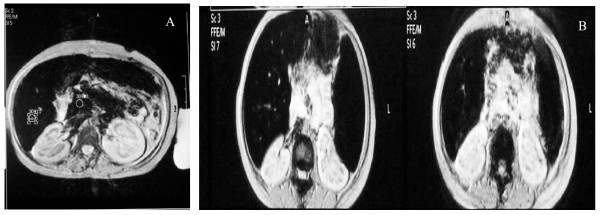
T2*-weighted GRE image of (A) splenectomized and (B) non splenectomized diabetic β-thalassemia major patients: (A) T2*-weighted GRE image (TR/TE/FA 120/15/20) of 12 yr-old female splenectomized β-thalassemia major with newly diagnosed diabetes showing low-signal intensity in the liver and the pancreas suggesting severe iron overload. (B) T2*-weighted GRE image (TR/TE/FA 120/15/20) of 16 yr-old female non splenectomized diabetic β-thalassemia major patient showing low-signal intensity in the liver and the spleen and normal signal intensity in the pancreas (TR, repetition time; TE, echo time; FA, flip angle).

**Figure 3:**
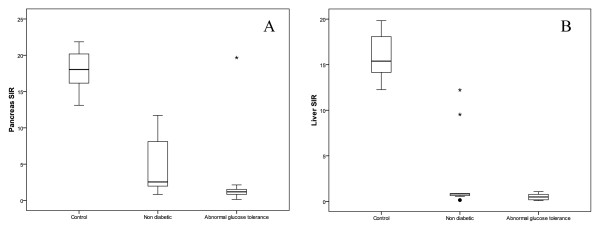
**Box and Whisker plot of signal intensity ratio of the pancreas (A), and the liver (B) of controls, thalassemic patients with normal and abnormal glucose tolerance**. The lines in the box represent the median, the boxes are the 25-75^th ^percentile range, and the whiskers are the 10-90^th ^percentile range.

Siderosis was detected on the basis of decreased signal in T2* GRE sequence to less than mean value ± 2 SD of normal controls (Figures 1, 2). Hepatic siderosis was diagnosed in 36/40 (90%) of thalassemic patients and pancreatic siderosis in 34/40 (85%) of patients. Siderosis was detected as early as the age of 8 years. The mean SIR of the liver and the pancreas were significantly reduced in different groups of patients compared to controls (P < 0.0001) (Table 3, Figure 3). Thalassemic patients with abnormal glucose tolerance showed significant reduction in SIR of the liver and the pancreas compared to those with NGT (P < 0.0001) with no significant difference between diabetics and IGT patients (P > 0.05). Spearman's correlation coefficient revealed that both SIR of pancreas and liver were correlated together (P < 0.001) and with age at diagnosis (P < 0.001, P < 0.01 respectively). Both hepatic and pancreatic SIR showed a negative correlation with liver enzymes, blood glucose and serum ferritin (P < 0.05) (Table 4).

**Table 3:** Comparison between different groups of thalassemic patients and controls as regards signal intensity ratio of the liver and the pancreas.

SIR of Liver	N	Median	IQR	Χ^2 ^value^#^	P value
**I) Controls**	20	15.389	14.158-18.074	42.828	<.001*
		
**II) Non diabetic(NGT)**	20	.833	.646-.861		
		
**III) abnormal glucose tolerance**	20	.489	.159-.769		

I/II) z value** = -5.423 P value < .001* I/III) z value** = -5.422 P value < .001* II/III) z value** = -2.379 P value = .017*					

**SIR of pancreas**	**N**	**Median**	**IQR**	**Χ^2 ^value^#^**	**P value**

**I) Controls**	20	18.036	16.165-20.183	38.384	<.001*
		
**II) Non diabetic(NGT)**	20	2.548	1.968-8.111		
		
**III) abnormal glucose tolerance**	20	1.185	.833-1.519		

I/II) z value** = -5.425 P value < .001* I/III) z value** = -5.423 P value < .001* II/III) z value** = -2.762 P value = .006*					

#Kruskal Wallis test; **Mann Whitney test;*Significant; IQR, interquartile range (25-75th percentile range).

**Table 4:** Correlation between signal intensity ratio of the liver, the pancreas and different studied parameters.

Parameters	SIR of liver		SIR of pancreas	
	
	*ρ *value^#^	P value	*ρ *value^#^	P value
**Age**	.065	.620	.088	.504

**Age at diagnosis**	.433	.005*	.542	<.001*

**Disease duration**	.228	.157	.279	.081

**Transfusion frequency**	-.162	.319	-.211	.192

**Ferritin**	-.409	.009*	-.532	<.001*

**FBG**	-.449	<.001*	-.416	<.001*

**PP**	-.514	<.001*	-.540	<.001*

**AST**	-.363	.021*	-.484	.002*

**ALT**	-.242	.132	-.411	.009*

**SIR of pancreas**	0.93	<.001*		

#Spearman rho correlation coefficient *Significant; N = 40; AST, aspartate aminotransferase; ALT, alanine aminotransferase; FBG, fasting blood glucose; PP, postprandial blood glucose; SIR, signal intensity ratio

Splenectomized thalassemic patients showed significant reduction in SIR of the pancreas compared to non splenectomized patients (P < 0.05) although insignificant difference was shown between both groups as regards age, duration of disease, serum ferritin, frequency of blood transfusion and SIR of the liver (P > 0.05) (Table 5).

**Table 5:** Comparison between non splenectomized and splenectomized thalassemic patients.

Parameters	non splenectomized (8)		Splenectomized (32)		t*	P	Sig.
				
	Mean ± SD		Mean ± SD				
Age (yrs)	12.63 ± 3.07		14.34 ± 3.30		1.34	0.19	NS

Duration of disease (yrs)	11.47 ± 3.65		13.50 ± 3.12		1.60	0.12	NS

Serum ferritin (mg/dl)	2374.63 ± 1183.90		2823.22 ± 1346.84		0.86	0.39	NS

Frequency of Blood Transfusion (wk)	3.13 ± 0.64		3.19 ± 0.69		0.23	0.82	NS

	**Median**	**IQR**	**Median**	**IQR**	Z**	**P**	**Sig.**

SIR of liver	0.679	0.451-0.967	0.707	0.273-0.857	-.135	0.89	NS

SIR of pancreas	2.95	2.047-15.562	1.743	1.014-3.211	-2.03	.042	S

*Student t test **Mann-Whitney test; SD, standard deviation; IQR, interquartile range; SIR, signal intensity ratio

## Discussion

The prevalence of IGT and diabetes in thalassemia major patients varied from 8% up to 27% [5,17-20] in various studies. Higher serum ferritin level in our patients with abnormal glucose tolerance compared to patients with NGT is in agreement with others [20]. Serum ferritin is considered as a risk factor for abnormal glucose tolerance in beta thalassemia patients [21]. Long term iron balance, rather than current iron status, is related to the development of glucose intolerance [22]. More compliant patients to chelation were encountered in the thalassemic group with NGT compared to the abnormal glucose tolerance group in our study.

Liver to noise ratio has been proposed to serve as a reference to quantify significant iron overload at 1.5 T [16]. The liver to noise ratio was slightly better correlated to liver iron concentration than was the liver to muscle ratio [23]. Gradient-echo sequences are particularly sensitive to iron deposition because of the lack of the 180° refocusing pulse that partially recovers signal loss from the field inhomogeneity in spin-echo images [24]. In the current study, the single breath T2* gradient-echo sequence was also preferred for its short scanning time making it more convenient in young age.

In the present study, there is significantly lower SIR of the liver and the pancreas in thalassemic patients compared to controls; the diagnosis of hepatic siderosis was posed in 36/40 patients (90%) and of pancreatic siderosis in 34/40 patients (85%) signifying that iron deposition in the pancreas is common and of variable degree since childhood. Several studies reported that pancreatic hemosiderosis results in abnormal MRI signal intensity, and there is iron overload in the pancreas in up to 75-100% of thalassemia major cases [11,13,25-28].

Lower SIR of the pancreas in thalassemic patients with abnormal glucose tolerance compared to non diabetic thalassemic patients is in agreement with Papakonstantinou et al. [13]. The SIR was correlated with endocrine function as assessed by OGTT [13] but contradict Midiri et al. [11]. In the study of Au et al. [25], the incidence of abnormal T2* was 81% among both diabetic and non diabetic thalassemic patients. Midiri et al. [11] suggested that markedly hypointense SI changes corresponding to iron deposition is seen first followed by a hyperintense pattern corresponding to a progressive fatty replacement of the pancreatic parenchyma. This may explain why our patients with IGT have slightly lower SIR of the pancreas than patients with overt diabetes.

Moreover, the lack of significant difference between diabetic and IGT patients in SIR of the liver and the pancreas may be explained that other causative factors for diabetes such as genetic predisposition and immune damage are unlikely to be reflected by MRI results [29]. Although established diabetic thalassemic patients seldom recover normal glucose tolerance [30]; impaired glucose tolerance is considered a reversible situation in beta-thalassemia [2,5].

We observed positive correlation between both pancreatic and hepatic siderosis and age at diagnosis, however, no correlation was revealed with age of patients. Au et al. [25] reported increase in pancreatic T2* values with age. Christoforidis [9] study showed a significant negative correlation between MRI values in liver and age.

Negative correlation detected between serum ferritin and SIR of the pancreas in our study is similar to Midiri et al. [11] but contradict others [12,13]. Argyropoulou et al [12] explained the lack of correlation between pancreatic siderosis and serum ferritin by the fact that T2 relaxation time depends on both siderosis and fatty infiltration of the pancreas reported in their adult group of patients. In addition, negative correlation between serum ferritin and SIR of the liver in our studied patients was in concordance with previous published data [12,13,15,31,32]; while no similar correlation was noted in other studies [9,33].

Reduced SIR of the liver in thalassemic patients with abnormal glucose tolerance compared to non diabetic patients was in agreement with Papakonstantinou et al [13]. This may denote the importance of hepatic iron deposition in the development of insulin resistance [21]. Iron overload and liver damage were the most important factors responsible for endocrine complications [4]. A strong correlation between pancreatic and hepatic siderosis was evident in our young studied patients. This observation was concordant with Brewer et al [34] who reported positive correlation between pancreatic R2* and hepatic iron content, although there was no similar correlation in previous reports [13,25,28,35]; this discrepancy may be attributed to pancreatic fatty replacement in their adult patients altering SIR of the pancreas.

Preliminary results of this study showed significant reduction in SIR of the pancreas in splenectomized thalassemic patients compared to those with intact spleen. This observation requires confirmation by further longitudinal studies and may reflect decreased extrahepatic iron buffering capacity in splenectomized patients [34] with accelerated iron deposition in the pancreas. The spleen acts as a store for nontoxic iron, thereby protecting the rest of the body from this iron [36]. Thus, splenectomized beta thalassemia major patients should be strictly monitored for pancreatic iron overload by MRI to avoid pancreatic dysfunction.

The present study advantages were inclusion of younger patients and performance of examinations at a single center. Study limitation included the lack of histological confirmation to verify the pancreatic MRI findings, and the over representation of poorly chelated patients which is common finding in developing countries. However, it is likely that advances in MRI techniques in iron assessment and chelation therapy will impact on future diabetic risk in thalassemia.

## Conclusions

Pancreatic siderosis could be detected by T2*-weighted gradient-echo MRI in young thalassemic patients, and was more evident in patients with abnormal glucose tolerance. After splenectomy, iron deposition may be accelerated in the pancreas. We recommend intensive chelation regimen to patients with IGT and NGT with reduced pancreatic SIR with strict follow up by MRI to assess improvement of pancreatic siderosis with reduction in markers of iron overload.

## Competing interests

The authors declare that they have no competing interests.

## Authors' contributions

RMM conceived the study, participated in its design and coordination and drafted the manuscript. KEA carried out and interpreted the MRI studies. AMS collected the clinical data of the children. All authors read and approved the final manuscript.
